# Impact of COVID-19 on the Development of Femoral Head Avascular Necrosis: A Systematic Review

**DOI:** 10.3390/medsci14030372

**Published:** 2026-07-03

**Authors:** Tomasz Poboży, Kamil Poboży, Julia Domańska-Poboża, Wojciech Konarski

**Affiliations:** 1 Department of Orthopedic Surgery, Medicover Hospital, 02-972 Warsaw, Poland; tomasz.pobozy@onet.pl; 2 Department of Neurosurgery, Brodnowski Masovian Hospital, 03-242 Warsaw, Poland; pobozykamil@gmail.com; 3 Department of Rheumatology, National Institute of Geriatrics, Rheumatology and Rehabilitation, 02-637 Warsaw, Poland; julia-domanska03@wp.pl; 4 Medical Rehabilitation Center, Sobieskiego 47D, 05-120 Legionowo, Poland

**Keywords:** femoral head avascular necrosis, COVID-19, corticosteroids, osteonecrosis, hip pathology

## Abstract

Background: COVID-19 has been linked to musculoskeletal complications, including femoral head avascular necrosis (AVN). Both COVID-19–related hypercoagulability and corticosteroid therapy have been proposed as contributing factors. This systematic review synthesizes current evidence on the occurrence, clinical characteristics, timing, and risk factors for femoral head AVN following COVID-19. Methods: A PRISMA-compliant systematic search of PubMed, Embase, and Scopus identified observational studies and case series (≥10 patients) reporting femoral head AVN in adults or adolescents with confirmed COVID-19. Data on epidemiology, symptom onset, imaging findings, and corticosteroid exposure were narratively synthesized due to heterogeneity. Results: Fifteen eligible studies described patients with post-COVID femoral head AVN. Symptom onset ranged from days to >12 months after infection. Early MRI often revealed asymptomatic or low-grade disease. Corticosteroid exposure was common and strongly associated with AVN severity; however, several studies reported AVN in patients without steroid use, whether this reflects an independent contribution of COVID-19 or unrecognized confounding cannot be determined from the available uncontrolled data. Higher cumulative steroid doses, severe pulmonary involvement, and elevated inflammatory markers were consistently linked to more advanced AVN stages. Conclusions: Femoral head AVN is an emerging post-COVID complication with variable timing and presentation. Corticosteroid exposure remains the principal risk factor; whether COVID-19 contributes independently of corticosteroids is unproven, and current evidence supports an association rather than a causal relationship. Awareness of this potential complication is warranted, although the role of early MRI screening remains to be established in prospective studies.

## 1. Introduction

The coronavirus disease 2019 (COVID-19) pandemic, caused by the novel severe acute respiratory syndrome coronavirus 2 (SARS-CoV-2) virus, has swept across the globe with over 775 million confirmed infections and more than 7 million deaths [1,2,3]. Besides its acute respiratory manifestations, COVID-19 is now recognized to have far-reaching systemic effects. The virus can trigger a hyperinflammatory and hypercoagulable state that damages organs beyond the lungs [4,5]. Among the emerging post-COVID complications is avascular necrosis (AVN)—the ischemic death of bone tissue leading to joint collapse. AVN (also known as osteonecrosis) classically affects weight-bearing joints (most often the femoral head of the hip) and has well-known risk factors such as high-dose corticosteroid use, trauma, alcohol abuse, and certain systemic diseases [1,6,7,8,9]. Early-stage AVN may be asymptomatic, but advanced stages cause chronic joint pain, limited mobility, and can necessitate surgical intervention [10]. In the context of COVID-19, concerns about AVN first arose from the extensive use of corticosteroids to treat severe COVID-19 pneumonia—a life-saving therapy with the potential side effect of steroid-induced osteonecrosis. Indeed, lessons from the 2003 SARS outbreak showed that prolonged corticosteroid therapy could precipitate AVN of the femoral head, raising concerns for the COVID-19 era [11,12]. Furthermore, COVID-19 itself may predispose patients to bone ischemia through thromboembolic complications; the virus-induced coagulopathy and endothelial dysfunction can compromise bone blood supply even in the absence of steroids [13,14]. Early in the pandemic, orthopedic specialists warned of a possible increase in AVN occurrence following COVID-19, anticipating a surge of osteonecrosis cases that could burden musculoskeletal care. Subsequent studies have borne out some of these fears, suggesting that COVID-19 infection is associated with an increased risk of developing AVN. An increasing number of reports and case series have documented AVN developing in patients during or after COVID-19 infection [11]. A recent systematic review compiled data from 13 studies encompassing 795 COVID-19 patients who developed avascular necrosis, with a mean age of approximately 46 years and a notable male predominance (66%). The majority of cases have involved the hip (femoral head), often presenting with hip pain and difficulty walking, though other joints (knees, shoulders, and others) have occasionally been affected as well [1]. The onset of AVN symptoms after COVID-19 is variable—reported intervals range from as early as about 2 weeks to as long as 14 months post-infection [1]. Steroid-induced interruption of bone blood flow and repair mechanisms is a likely driver of osteonecrosis in this setting [15,16]. However, importantly, not all post-COVID AVN cases have a history of steroid use. For example, in one series from India, 60% of the patients who developed AVN after COVID-19 had not received any corticosteroids [17]. This observation has been interpreted as implicating COVID-19 severity and its pathophysiological effects—such as hypercoagulability, severe inflammation, and hypoxic damage—in AVN development. Hypercoagulable states are themselves an established cause of femoral head AVN: antiphospholipid syndrome (APS), a prototypic thrombophilic disorder, can produce femoral head osteonecrosis even in the absence of corticosteroid exposure [18]. This provides a mechanistic precedent for a possible thrombotic contribution of COVID-19–associated coagulopathy.

However, AVN also occurs in steroid-naive individuals independently of COVID-19, and pre-existing asymptomatic osteonecrosis, unrecognized risk factors, and selection bias cannot be excluded; these data therefore support an association rather than an independent causal role. It also highlights that even patients with mild COVID-19 (who didn’t require steroids) are not entirely exempt from this complication, though most reported cases did occur in moderate to severe COVID-19 illness requiring hospitalization [1,19,20,21,22].

The potential link between COVID-19 and avascular necrosis has important clinical implications as the world continues to challenge with the long-term fallout of the pandemic. With millions of COVID-19 survivors at risk, it is critical to understand how and why AVN develops in this context. Accordingly, we have undertaken a systematic review to synthesize the current evidence on COVID-19-associated AVN of the femoral head, including evidence on its occurrence and epidemiology, describing the clinical characteristics, timing of its presentation after COVID-19, and identifying potential risk factors, with particular attention to corticosteroid exposure and disease severity.

## 2. Materials and Methods

This systematic review was conducted in accordance with the Preferred Reporting Items for Systematic Reviews and Meta-Analyses (PRISMA) guidelines [23] and followed a predefined protocol registered prospectively in PROSPERO (registration number: CRD420251166498).

### 2.1. Eligibility Criteria

Studies were eligible for inclusion if they met the following criteria: human research involving adults or adolescents aged 16 years or older (patients aged 16 years or older were included because, by this age, the femoral head is approaching skeletal maturity and the pathophysiology, imaging diagnosis, and staging of AVN are comparable to those in adults) with confirmed COVID-19 (COVID-19 was considered confirmed according to the diagnostic criteria of each included study (reverse-transcription PCR and/or rapid antigen testing, or a clinical and radiological diagnosis where defined by the primary authors); observational study design (cohort, case–control, or cross-sectional) or case series including ≥10 patients; and assessment of AVN of the femoral head. Only articles published in English from 1 December 2019, to the 10 November 2025, were considered. Exclusion criteria included case reports, small case series with fewer than 10 participants, reviews, commentaries, conference abstracts, non-human experiments, studies lacking confirmed COVID-19 diagnosis, and studies reporting AVN at sites other than the femoral head. The ≥10-patient threshold was applied to ensure a minimum level of data reliability, as smaller reports are subject to extreme selection bias—disproportionately capturing atypical or severe presentations—and do not permit meaningful assessment of incidence, risk factor distribution, or staging patterns.

### 2.2. Information Sources and Search Strategy

A comprehensive search was performed in PubMed, Embase, and Scopus, covering the period from 1 December 2019, to the 10 November 2025 (final search). Search terms combined controlled vocabulary and free-text expressions related to COVID-19 and avascular necrosis. Example queries included combinations of “COVID-19”, “SARS-CoV-2”, “avascular necrosis,” “osteonecrosis”, and “femoral head necrosis.” Full search strategies for all databases were defined a priori in the protocol and are listed in Appendix A. Reference lists of relevant articles were also screened to identify additional eligible studies.

### 2.3. Study Selection

Two reviewers independently screened titles and abstracts, applying the eligibility criteria. Full texts of potentially relevant studies were subsequently assessed. Any discrepancies were resolved through discussion and, if necessary, consultation with a third reviewer to reach consensus. Reference management and deduplication were performed using EndNote 19.

### 2.4. Data Extraction

Data were extracted independently by two reviewers using a standardized template developed in Microsoft Excel. Extracted variables included study characteristics (authors, year, country, design, sample size), participant characteristics (age, sex, comorbidities, severity of COVID-19, corticosteroid exposure), AVN characteristics (anatomical site, stage, and time from COVID-19 to onset), and epidemiological outcomes such as incidence or prevalence. Information on progression and reported treatments was also collected when available.

### 2.5. Data Synthesis

Due to the anticipated heterogeneity in study design, diagnostics, and reporting of AVN outcomes, findings were synthesized narratively. Descriptive summaries were generated, focusing on epidemiological patterns, clinical presentation, and potential risk factors. Where studies provided comparable quantitative data, incidence or prevalence estimates were tabulated for contextual interpretation rather than pooled statistically. For synthesis, studies were grouped according to their primary objective: those estimating the occurrence or risk of AVN in defined COVID-19 populations were considered separately from those characterizing the features of patients with already-established post-COVID AVN, as these designs address different questions.

### 2.6. Risk of Bias Assessment

The methodological quality and risk of bias of the included studies were assessed using design-specific tools. Cohort studies were evaluated using the Newcastle–Ottawa Scale (NOS) for cohort studies [24]. Cross-sectional studies were assessed using NOS-xs, an adaptation of the NOS developed for cross-sectional studies [25]. Case series were assessed using the Joanna Briggs Institute Critical Appraisal Checklist for Case Series [26].

## 3. Results

**Figure 1:**
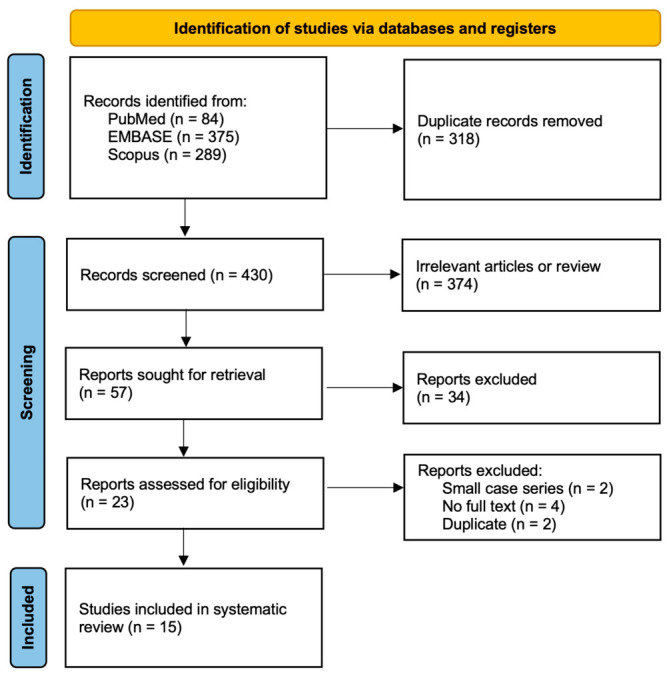
Flow diagram of literature search [23].

This systematic review included data from 15 distinct studies. The detailed process of literature selection, including inclusion and exclusion criteria, is illustrated in the flow diagram (Figure 1). Baseline characteristics of the included studies are summarized in Table 1.

### 3.1. Occurrence and Epidemiology

This section summarizes studies that estimated the occurrence or risk of femoral head AVN in defined COVID-19 populations.

A prospective observational study by Ali et al. [27] evaluated the occurrence of femoral head AVN among 110 patients who received corticosteroid-based COVID-19 therapy and subsequently reported hip discomfort. All participants underwent MRI within six months of treatment. AVN was detected at early stages: 4.5% had Stage I changes, 2.7% had Stage II, and 1.1% had Stage III, with no Stage IV cases identified. The study indicates that although AVN is uncommon, it represents a clinically relevant complication following COVID-19 therapy, particularly in younger patients presenting with hip symptoms.

In the cross-sectional study by Koutalos et al. [31], 80 hospitalized COVID-19 patients were evaluated, including 40 who received corticosteroids and 40 who did not. All patients underwent MRI of the hips, shoulders, and knees, and clinical assessment with the Oxford Hip Score. Three patients in the corticosteroid-treated group (3/40; 7.5%) were diagnosed with femoral head AVN, while no cases occurred in the non-steroid group. Pearson chi-squared test indicated a trend towards significant difference between the two groups regarding hip osteonecrosis (x^2^ = 3.1, *p* =0.07, odds ratio = 1.08, 95% CI: 0.99–1.18).

In the study by Okewunmi et al. [32], analysis of a national database demonstrated that the incidence of AVN among patients undergoing total hip arthroplasty increased during the COVID-19 pandemic. AVN accounted for 1.6% of THAs performed in 2020–2021 compared with 1.4% in 2016–2019. In a cohort from April 2020 to December 2021, patients with a prior COVID-19 diagnosis had a higher prevalence of femoral head AVN (3.9%) than those without COVID-19 (3.0%). These findings indicate that a history of COVID-19 infection was associated with an increased likelihood of developing osteonecrosis.

In the study by Takashima et al. [36], 41 hospitalized patients treated for COVID-19–related pneumonia or other COVID-19 complications were evaluated for the occurrence of femoral head AVN. After one in-hospital death, 40 patients were included in the analysis. None of the patients reported hip pain during follow-up. MRI screening was performed in 26 patients at a mean of 3 months after discharge, and asymptomatic AVN was detected in one individual (3.8%). The affected patient was a 57-year-old man with moderate COVID-19 pneumonia who received dexamethasone equivalent to 400 mg of prednisolone. This case remained asymptomatic but showed radiologic progression on serial imaging.

### 3.2. Clinical Characteristics

This section summarises studies describing the characteristics of patients with already-diagnosed post-COVID femoral head AVN.

In the study by Assad et al. [28], a single-center retrospective case series described 17 patients who developed femoral head AVN following COVID-19 infection over a two-year period (January 2021–December 2022). The mean age was 38.6 years, and most patients were male (70.6%). The average BMI was 28.3 kg/m^2^. Nearly half of the patients (47%) had been hospitalized for COVID-19, although none required ICU admission. Corticosteroid exposure during the infection was common, reported by 64.7% of patients. The mean interval between COVID-19 infection and clinical presentation was 6.5 months. Bilateral hip pain was the predominant symptom (82.3%), and limping was present in 47% of cases. MRI confirmed AVN in all patients, with grades ranging from 2 to 4.

In the study by Goel et al. [29], a retrospective analysis was conducted on ten patients who had been hospitalized with COVID-19 and later developed hip pain. MRI confirmed AVN of the femoral head in all cases. The mean age was 61 years, with six women and four men. AVN was right-sided in six patients and left-sided in four. The course of COVID-19 was mild in three patients, moderate in five, and severe in two. Pain severity was high, with VAS scores ranging from 6 to 9 (mean: 7.7). On MRI, AVN severity according to the Steinberg scale was distributed as follows: Stage 2 in six patients, Stage 3 in one patient, and Stage 4 in three patients.

In the study by Imagama et al. [30], the authors analyzed 5371 patients with femoral head AVN from a large multicentre Japanese registry and identified 20 patients (32 hips) who developed femoral head AVN after COVID-19. These patients were compared with 693 individuals (1197 hips) who developed corticosteroid-associated femoral head AVN unrelated to COVID-19. The COVID-19 group showed a clear male predominance (15 men, 5 women) compared with the corticosteroid group. The mean age in the COVID-19 group was 48 years, similar to the comparator group. Bilateral involvement occurred in 60% of COVID-19 related cases. JIC type distribution in the COVID-19 group showed that 78.2% of hips were classified as type C2, compared to 51.2% in the corticosteroid-associated group. Stage distribution ranged from stage 1 to stage 4, with no significant differences compared with the corticosteroid group (*p* = 0.20).

In the cross-sectional study by Koutalos et al. [31], COVID-19 patients who received corticosteroids and developed femoral head AVN (3/40) showed higher inflammatory markers compared with those who did not develop AVN (37/40), including elevated white blood cell counts (9200 vs. 7000/μL), ferritin levels (1961 vs. 997 ng/mL), and C-reactive protein (8.2 vs. 3.3 mg/dL).

In the study by Sehrawat et al. [33], a retrospective cross-sectional analysis was performed on 50 patients who developed non-traumatic femoral head AVN following COVID-19 infection. The mean age was 36.3 years, and the cohort consisted predominantly of men (45/50). Bilateral involvement was common (78%). COVID-19 severity varied, with 60% managed at home and the remainder requiring ward or ICU care. The study evaluated correlations between AVN characteristics and COVID-19 variables, including symptom duration, severity, time interval to onset of hip pain, and steroid intake. AVN stage showed a statistically significant association with COVID-19 severity.

In the study by Sharma et al. [34], 25.6% of patients diagnosed with avascular necrosis had a prior history of COVID-19 infection. COVID-19 status showed statistically significant associations with age distribution, comorbidities, and timing of symptom onset, indicating that a substantial subset of AVN cases occurred in individuals recovering from COVID-19. Patterns of hip or back pain, by contrast, did not differ significantly between COVID-positive and COVID-negative patients.

In the study by Sujir et al., 24 patients (35 hips) developed femoral head AVN after recovering from COVID-19. The mean age was 36.6 years, and 58.3% of participants were male. Hip involvement was bilateral in 45.8% of cases, right-sided in 29.2%, and left-sided in 25%. Comorbidities were present in 33.3% of participants, and the interval from COVID-19 infection to symptom onset was shorter in patients with diabetes and hypertension (3 months) compared with those without comorbidities (7.1 months).

In the study by Velchov et al. [37], 24 patients with COVID-19 pneumonia were evaluated for femoral head AVN. The mean age of the patients was 56 ± 15 years. Eight patients had a moderate course of infection, while sixteen had severe disease. Severe COVID-19 cases showed higher-grade AVN and greater pain levels than moderate cases (*p* < 0.05). Laboratory values at orthopedic assessment showed elevated D-dimer and CRP levels, consistent with ongoing systemic inflammation.

In the retrospective study by Seong et al. [38], which included 84 patients with femoral head AVN, ages ranged from 24 to 73 years with a median age of 39.5 years. The male-to-female ratio was 2.7:1. BMI ranged from 18.4 to 39.2 kg/m^2^, with a median of 24.8 kg/m^2^, and nearly half of the cohort (48.8%) fell within the normal weight range. Most patients were classified as ARCO stage 2 (57 patients, 67.9%), and bilateral femoral head involvement was identified in 50 patients (59.5%).

In the study by Dhanasekararaja et al. [40], 22 consecutive patients (39 hips) who developed femoral head AVN after recovering from COVID-19 were evaluated between November 2020 and October 2021. The cohort consisted of 20 males and 2 females, with a mean age of 38.8 years (range 20–74) and a mean BMI of 27.6 ± 4.4. Radiologic assessment showed that 14 hips (35.9%) had large necrotic lesions corresponding to Kerboul grades 3 or 4, and 28 hips (71.8%) demonstrated JIC type C involvement. Most hips (36 of 39) exhibited features consistent with classical AVN. Plain radiographs typically revealed subchondral cysts in the weight-bearing dome and lateral femoral head, while MRI showed the characteristic focal serpentine line and double-line sign in all cases, with no acetabular involvement.

In the study by Hogea et al. [41], 32 patients diagnosed with femoral head AVN between August 2022, and January 2024 were evaluated to explore whether COVID-19 infection contributes to AVN development independently of steroid exposure. The mean age was 53.8 ± 9.6 years, and 56.3% of the cohort were male. Comorbidities were common, including hypertension (43.8%), diabetes (34.4%), and obesity (31.3%). Nineteen patients (59.4%) had confirmed COVID-19 infection, while 13 (40.6%) were COVID-negative. Among COVID-positive patients, 73.7% received corticosteroids with antiviral therapy, and 68.4% demonstrated pulmonary involvement. Bilateral AVN was observed in three patients, all of whom had COVID-19 and severe pulmonary disease.

### 3.3. Timing of Presentation of Femoral Head AVN After COVID-19

Across the included studies, the interval between COVID-19 infection and the onset of femoral head AVN symptoms varied widely (Table 2). Most frequently, symptoms emerged within several months after infection, although earlier and delayed presentations were also reported. In Assad et al. [28], over half of patients developed symptoms within 2–6 months, while Goel et al. [29] and Velchov et al. [37] reported much earlier onset, occurring within weeks. Several studies demonstrated later presentation, with median or mean intervals of 6–12 months (Koutalos et al. [31], Sehrawat et al. [33], Sujir et al. [35], Agarwala et al. [39], Dhanasekararaja et al. [40]). Isolated early cases were also identified, such as the 1-month onset reported by Takashima et al. [36]. Reported intervals should be interpreted cautiously, as the included studies variably referred to symptom onset, the time of MRI diagnosis, or first clinical presentation, and several reported a single composite interval. Overall, symptom onset ranged from within a few weeks to more than 12 months after COVID-19. The shortest reported intervals—notably the 7–22-day window in Goel et al.—are biologically implausible for newly developed osteonecrosis and most likely represent pre-existing, previously asymptomatic disease detected after infection rather than de novo necrosis.

### 3.4. Corticosteroid Exposure

Corticosteroid exposure varied substantially across studies, both in cumulative dose and treatment duration (Table 3). Mean total corticosteroid doses ranged from moderate regimens of approximately 400 mg methylprednisolone equivalent (Takashima et al. [36]) to higher exposures exceeding 840 mg prednisolone equivalent (Agarwala et al. [39]) and 811 mg methylprednisolone equivalent (Dhanasekararaja et al. [40]). Studies such as Velchov et al. [37] reported prolonged courses with combined dexamethasone and methylprednisolone therapy, while others included mixed regimens or incomplete dose reporting. Despite heterogeneity, most cohorts demonstrated substantial steroid use during COVID-19 management, highlighting corticosteroid exposure as a consistent factor among patients who later developed femoral head AVN.

Interestingly, in the study by Imagama et al. [30] patients in the COVID-19 group had markedly shorter corticosteroid exposure (mean 2.3 months vs. 44.9 months; *p* < 0.0001) and lower maximum daily doses (mean 28.1 ± 19.8 mg vs. 53.2 ± 117.3 mg) (*p* = 0.011), and fewer received corticosteroid-pulse therapy.

A cross-sectional study by Koutalos et al. [31] compared COVID-19 patients who received corticosteroids and developed femoral head AVN (3/40) with those who did not (37/40). Patients with osteonecrosis received substantially higher cumulative corticosteroid doses (median 2200 mg vs. 560 mg), were treated for a longer duration (median 29 vs. 21 days), and had longer hospital stays (median 15 vs. 6 days).

In the study by Sehrawat et al. [33] most patients had received corticosteroids during their illness: 22% received both injectable and oral steroids, 16% intravenous steroids only, and 46% oral steroids; 16% recovered without steroid exposure.

In the study by Takashima et al. [36], among 41 patients, 29 (71%) received corticosteroids and 1 patient developed femoral head AVN.

In the study by Seong et al. [38] The group treated with dexamethasone and methylprednisolone compared to dexamethasone alone had more extensive COVID-19 lung involvement (60% vs. 30%) and experienced longer hospital stay in both the general ward (14.2 vs. 10.6 days; *p* = 0.018) and ICU (5.5 vs. 2 days; *p* = 0.020). This group also received longer steroid courses (19.3 vs. 12.3 days) and significantly higher cumulative dexamethasone-equivalent doses (380 mg vs. 125 mg; *p* < 0.001). AVN symptoms emerged earlier in the dexamethasone + methylprednisolone group than in the dexamethasone-only group (7.5 vs. 12 months; *p* = 0.004). In multivariable analysis, cumulative steroid dose was the only independent predictor of AVN severity (OR 1.015, 95% CI: 1.001–1.028, *p* = 0.032), and ARCO stage 3 patients received higher cumulative doses than stage 2 patients (240 vs. 126 mg).

Comparative analysis by Hogea et al. [41] showed that COVID-positive patients were significantly more likely to receive steroid treatment (73.7% vs. 15.4%) and to have pulmonary involvement (68.4% vs. 15.4%) than COVID-negative patients. However, the incidence of AVN did not differ significantly between the two groups. Logistic regression analysis adjusting for age, sex, and comorbidities found that COVID-19 infection was not independently associated with AVN (OR 1.28, 95% CI 0.42–3.90), whereas steroid therapy was a significant predictor (OR 5.21, 95% CI 1.50–18.06).

### 3.5. Risk of Bias Assessment and Methodological Heterogeneity

The results of the quality appraisal are presented in Table 4. Overall, the methodological quality of the included studies was moderate. Most studies were rated as having moderate quality/moderate risk of bias, while two studies were classified as low quality/high risk of bias and two as high quality/low risk of bias. This pattern reflects the predominantly retrospective and observational nature of the available evidence, as well as the frequent lack of control groups and limited adjustment for confounding.

The methodological characteristics of the included studies are summarised in Table 5. The studies varied considerably in their primary objective and design, the AVN staging system applied (ARCO, Steinberg, JIC, Kerboul, or non-standardised grading), the imaging modality and its timing relative to COVID-19, the duration of follow-up, and the way COVID-19 severity was defined. This heterogeneity precludes direct comparison of disease frequency, stage distribution, and timing across studies, and was the principal reason for synthesising the evidence narratively rather than by meta-analysis.

## 4. Discussion

This systematic review synthesizes current evidence on the development of femoral head AVN following COVID-19 and highlights several important clinical patterns. Across the included studies, AVN emerged as a notable musculoskeletal complication, occurring in patients with a broad range of COVID-19 severity and with variable latency from infection to symptom onset. Although corticosteroid exposure remains a well-established risk factor and was common in many cohorts, a subset of patients developed AVN despite minimal or absent steroid therapy. This observation is compatible with a contribution of COVID-19 but does not establish one: pre-existing asymptomatic osteonecrosis, alcohol use, thrombophilic disorders, other unmeasured confounders, and detection bias cannot be excluded from the available studies. The current evidence therefore supports an association between COVID-19 and femoral head AVN rather than a causal relationship.

Several mechanistic and dose-threshold statements in this section are drawn from previously published narrative or systematic reviews rather than from the primary studies included here, and are attributed accordingly.

Post-COVID AVN of the femoral head appears to have a multifactorial pathogenesis. Corticosteroid therapy during acute COVID-19 is the strongest modifiable risk factor identified—high-dose or prolonged steroid use is well-known to precipitate osteonecrosis by disrupting bone microcirculation [42]. However, importantly, AVN cases have also been observed in COVID-19 survivors without any steroid exposure, indicating additional mechanisms beyond corticosteroids [43]. Several biologically plausible mechanisms have been proposed by which SARS-CoV-2 infection could, in principle, contribute to bone ischemia, including endothelial injury, microvascular thrombosis, and hyperinflammatory damage [4,44,45,46]. Endothelial dysfunction and microthrombus formation are documented features of COVID-19, and the associated cytokine response can promote hypercoagulability and impaired fibrinolysis [42]. Whether these processes actually produce femoral head ischemia and osteonecrosis in this context has not been demonstrated, and their proposed link to AVN should be regarded as hypothetical rather than established. Some authors have hypothesized that SARS-CoV-2 may act as an independent contributor to femoral head AVN via immune-mediated vascular injury, though this remains unproven in the absence of adequately controlled prospective data [43]. Thus, while steroid use remains a major precipitant, the pathogenesis of post-COVID AVN is multifactorial, involving a combination of steroid effects and COVID-related coagulopathy and inflammation.

Post-COVID femoral head AVN is characterized by the wide variability in latency from COVID-19 infection to onset of hip symptoms which is also visible in our review. Reported intervals range from mere days or weeks after recovery to well over a year. This diversity in time course suggests that the biological progression of osteonecrosis can differ substantially between patients. In steroid-associated cases, osteonecrosis often becomes symptomatic within a few months (one review focusing on high-dose steroid cases noted a mean onset of ~80 days) [47], whereas in other patients the damage may evolve subclinically for a year or more before causing pain. Such variability underscores the diagnostic challenge—clinicians must remain vigilant for AVN in post-COVID patients even long after the acute infection, as delayed or missed diagnoses have been reported. The consensus is to maintain a high index of suspicion for at least the first 6–12 months after moderate-to-severe COVID (and up to 3 years in some high-risk cases) [43], given that symptom onset may be insidious and significantly delayed.

Given the latent and often asymptomatic early course of osteonecrosis, early imaging surveillance may be warranted in selected post-COVID patients at elevated risk, though formal recommendations cannot be established based on current evidence. Notably, the onset of clinical symptoms frequently lags behind radiological changes in the femoral head [43]. Patients can have significant osteonecrosis on MRI while still minimal or no hip pain, especially in the initial stages. Relying solely on symptom-driven evaluation will therefore underestimate the true incidence of AVN. Several reports have advocated proactive MRI screening of the hips in high-risk COVID-19 survivors to catch subclinical AVN before collapse occurs [42]. In clinical practice, it has been suggested that patients who endured severe COVID-19 (especially with heavy steroid treatment) might benefit from scheduled hip MRI at 3, 6, or 12 months post-infection. This proposal is hypothesis-generating only: no prospective study has demonstrated that such screening improves outcomes, and any schedule would require validation before it could be recommended.

Evidence suggests a dose–response relationship between corticosteroid therapy for COVID-19 and subsequent AVN risk. High cumulative steroid doses, longer duration of therapy, and intensive regimens (such as sequential use of dexamethasone followed by methylprednisolone) have been linked to earlier onset and increased severity of AVN. Prior research has suggested that exceeding certain cumulative dose thresholds may substantially increase AVN risk—one analysis noted that methylprednisolone below approximately 5 g was associated with lower risk, whereas doses above 5–10 g appeared to correlate with higher rates of osteonecrosis; however, these thresholds were not derived from COVID-19 cohorts and their applicability to this population remains uncertain [47]. Similarly, a cumulative exposure around 2000 mg of prednisolone equivalent is often cited as a critical risk level for osteonecrosis [48]. In COVID-19 care, many patients with severe disease received high-dose steroids (e.g., 6 mg dexamethasone for 10 days, sometimes escalated to intravenous methylprednisolone) [49,50]. Unsurprisingly, such patients constitute the majority of AVN cases. A comprehensive review noted a clear correlation between steroid intensity and AVN: particularly, patients treated with both dexamethasone and methylprednisolone (or prolonged courses) were disproportionately represented among early-onset AVN cases [42]. Although individual susceptibility varies, higher steroid burden appears to accelerate osteonecrosis and may increase the likelihood of multi-focal or bilateral disease. These observations suggest that clinicians should aim to use the minimal effective steroid dose and duration for COVID-19, recognizing that the precise risk threshold in this population has not been established [51].

The above considerations have significant clinical implications for the post-COVID period. Physicians should maintain a low threshold to investigate persistent musculoskeletal symptoms in COVID-19 survivors—new or lingering hip pain after COVID-19 should prompt evaluation for possible AVN, especially in those who received corticosteroids. Although some authors have proposed targeted imaging of higher-risk survivors—for example, those with severe COVID-19 (ICU admission or a pronounced inflammatory response) or substantial corticosteroid exposure (high cumulative dose or multiple courses) [52]—the current evidence is insufficient to recommend routine or targeted MRI screening of asymptomatic patients. No prospective study has demonstrated that such surveillance improves outcomes or is cost-effective; its role therefore remains uncertain and requires evaluation in future prospective studies. Some authors recommend that any patient with known risk factors (such as an exceptionally high D-dimer or inflammatory markers during COVID-19, indicating a hypercoagulable state) be monitored closely for osteonecrosis. Whether scheduled hip imaging at around 3 months post-recovery would improve outcomes in high-risk patients is unknown and requires prospective evaluation; the rationale is that early imaging can detect silent AVN that symptom-based monitoring would miss [53]. Moreover, patients who had an extremely high inflammatory burden (reflected by markers like CRP, ferritin) might merit screening even if they did not receive steroids, since the intense cytokine storm and coagulopathy could independently predispose to AVN [42].

### Limitations

Our systematic review has several important limitations that reflect the nature of the currently available literature on COVID-19-associated AVN of the femoral head. Most included studies were retrospective case series or observational cohorts, often conducted without control groups and involving relatively small sample sizes. These methodological constraints limit the generalizability and validity of the findings. Also, our search was restricted to peer-reviewed published literature; trial registries and grey literature (such as conference proceedings, preprints, theses, and unpublished reports) were not searched. As a result, ongoing or unpublished data may have been missed, and the possibility of publication bias cannot be excluded. The handling of pre-existing osteonecrosis risk factors—including alcohol use, prior trauma, systemic inflammatory disease, and chronic corticosteroid therapy preceding COVID-19—varied across the included studies: some explicitly excluded such patients (for example, by excluding habitual alcohol use, prior hip trauma or surgery, autoimmune disease, and chronic steroid therapy), whereas others did not report or did not control for them. This inconsistency precludes attribution of AVN specifically to COVID-19 and represents an important source of residual confounding. Clinical and methodological heterogeneity among included studies was substantial and represents a central limitation of this review. AVN staging systems differed across studies—some used the ARCO classification, others the Ficat, Steinberg, or JIC systems—precluding direct comparison of disease severity between cohorts. Imaging protocols were equally inconsistent: MRI was performed at variable time points after COVID-19 recovery, ranging from weeks to over a year, meaning that studies captured different phases of the natural history of AVN. This variability in imaging timing alone may account for apparent differences in AVN prevalence and stage distribution between studies. COVID-19 severity was defined heterogeneously across cohorts, with some studies relying on WHO criteria, others on local institutional classifications, and several providing no formal definition at all. Corticosteroid regimens—arguably the most clinically critical variable—were inconsistently reported: not all studies specified the type of steroid used (e.g., dexamethasone vs. methylprednisolone), the route of administration, cumulative dose, or treatment duration, making cross-study comparisons of steroid exposure unreliable. Finally, follow-up durations differed widely, from a few months to over two years, introducing the risk that studies with shorter follow-up systematically underdetected later-onset AVN cases. Collectively, these sources of heterogeneity precluded meta-analytic pooling and necessitated a narrative synthesis, which inherently limits the strength of conclusions that can be drawn. These discrepancies introduce a high risk of bias and make it challenging to draw firm conclusions. Additionally, key confounding factors such as preexisting comorbidities (e.g., diabetes, obesity, hypercoagulability) were not consistently controlled or adjusted for in the analyses. Publication bias represents an additional and particularly relevant limitation in this emerging field. Given the novelty of the COVID-19–AVN association, studies reporting dramatic, severe, or bilateral presentations—such as rapid femoral head collapse in young patients—are disproportionately likely to be submitted and accepted for publication. Conversely, mild, asymptomatic, or early-stage AVN cases detected incidentally on MRI screening, as well as cases that resolved spontaneously or responded to conservative management, are substantially less likely to appear in the literature. This selective reporting may lead to an overestimation of AVN severity and incidence in the post-COVID population. Furthermore, the absence of a prospectively registered, population-based screening programme means that the true denominator—i.e., the number of COVID-19 survivors at risk—remains unknown, making it impossible to calculate reliable incidence rates from the currently available studies. These considerations reinforce that the reported burden of post-COVID femoral head AVN should be interpreted with caution, and that prospective cohort studies with systematic MRI screening are urgently needed to capture the full clinical spectrum of this complication.

These issues underscore the need for cautious interpretation of the current data and reinforce that the observed associations between COVID-19, corticosteroid use, and AVN should be considered as hypothesis-generating rather than conclusive. To advance our understanding, high-quality prospective research is urgently needed. Future studies should comprise prospective, preferably multicenter cohorts of COVID-19 survivors enrolling both corticosteroid-exposed and steroid-naive patients, with systematic MRI screening at standardized intervals; uniform use of a single staging system (e.g., ARCO) to enable cross-study comparison; standardized recording of cumulative corticosteroid exposure as prednisolone-equivalent doses; clearly defined denominators to permit true incidence estimates; and long-term, registry-based follow-up to capture late-onset and asymptomatic disease and to determine whether the observed association reflects a causal contribution of COVID-19 independent of corticosteroid therapy.

## 5. Conclusions

This systematic review demonstrates that femoral head avascular necrosis has emerged as a significant post-COVID complication with wide variability in onset, clinical presentation, and severity. Corticosteroid exposure remains the most consistent risk factor. AVN has also been documented in patients with minimal or no steroid use, which raises the possibility of a contribution from COVID-19 but does not establish one, as pre-existing asymptomatic disease, alcohol use, thrombophilia, unmeasured confounding, and detection bias cannot be excluded; the current evidence therefore supports an association rather than causation. The role of early MRI is uncertain: the available evidence is insufficient to recommend routine or targeted screening after COVID-19, and its value should be evaluated in prospective studies. Clinicians should maintain a high index of suspicion for AVN in patients with prior COVID-19—particularly those treated with steroids or reporting persistent hip pain. Further prospective studies are needed to determine whether the observed association reflects a causal contribution of COVID-19, to define true incidence and to establish evidence-based screening and management strategies in the post-COVID population.

## Figures and Tables

**Table 1:** Characteristics of Included Studies.

Study	Country	Design	Population
Ali et al. [27]	China	Prospective observational study	110
Assad et al. [28]	Iraq	Single-centre retrospective case series	17
Goel et al. [29]	India	Single-centre retrospective case series	10
Imagama et al. [30]	Japan	Multi-centre retrospective study	5371
Koutalos et al. [31]	Greece	Cross-sectional study	80
Okewunmi et al. [32]	USA	Retrospective study	1,127,796 total hip arthroplasties
Sehrawat et al. [33]	India	Cross-sectional study	50
Sharma et al. [34]	India	Cross-sectional study	86
Sujir et al. [35]	India	Prospective observational study	24
Takashima et al. [36]	Japan	Prospective observational study	41
Velchov et al. [37]	Bulgaria	Retrospective study	24
Seong et al. [38]	Uzbekistan	Retrospective study	84
Agarwala et al. [39]	India	Retrospective study	48
Dhanasekararaja et al. [40]	India	Prospective observational study	22
Hogea et al. [41]	Romania	Retrospective study	32

**Table 2:** Interval Between COVID-19 Infection and Onset of Femoral Head Avascular Necrosis Symptoms.

Study	Interval from COVID-19 and Onset of Symptoms
Assad et al. [28] (N = 17)	2–6 months—9 patients (53%) 7–12 months—7 patients (41%) >12 months—1 patient (9%)
Goel et al. [29] (N = 10)	Range 7–22 days (mean 14 days)
Koutalos et al. [31] (N = 3)	Median 265 days
Sehrawat et al. [33] (N = 50)	Mean 359 days
Sujir et al. [35] (N = 24)	Mean 6 months
Takashima et al. [36] (N = 1)	1 month
Velchov et al. [37] (N = 24)	Mean: 57 ± 12 days
Seong et al. [38] (N = 84)	Range 4–22 months (median 12 months)
Agarwala et al. [39] (N = 48)	Mean: 179 days
Dhanasekararaja et al. [40] (N = 22)	Mean 7.5 months

**Table 3:** Corticosteroid Exposure.

Study	Steroid Use	Duration of Steroid Use	Dose (as Reported)	Prednisolone-Equivalent Cumulative Dose (Estimated)
Assad et al. [28]	11/17 patients (64.7%)	7 days—3 patients (27.3%) 5 days—4 patients (36.4%) 4 days—3 patients (27.3%) 2 days—1 patient (9%)	ND	ND
Goel et al. [29]	4/10 patients (40%)	2 weeks	2 × 8 mg daily	NC (steroid not specified)
Imagama et al. [30]	20/20 patients (100%)	Mean: 2.3 ± 2.9 months	Mean maximum dose per day: 28.1 ± 19.8 mg	NC (maximum daily dose only)
Koutalos et al. [31]	3/3 patients (100%)	Median: 29 days	Median total prednisolone dose: 2200 mg	2200 mg (as reported)
Sehrawat et al. [33]	42/50 patients (84%)	Mean: 25 days	Mean total prednisolone dose: 865 mg	865 mg (as reported)
Sharma et al. [34]	14/22 patients (64%)	Median: 5 days	Mean total prednisolone dose: 51.7 ± 8.8 mg/day	NC (daily dose only)
Sujir et al. [35]	20/24 patients (83%)	ND	Mean total prednisolone dose: 713 mg	713 mg (as reported)
Takashima et al. [36]	1/1 (100%)	10 days	Mean total prednisolone dose: 400 mg	400 mg (as reported)
Velchov et al. [37]	24/24 (100%)	Mean 5 days for dexamethasone and 30 days for methylprednisolone.	Moderate infection (N = 8): dexamethasone 2 × 4 mg/day Severe infection (N = 16): dexamethasone 2 × 4 mg/day + methylprednisolone 3 × 40 mg/day	≈270 (moderate)—≈4800 (severe), regimen-based
Seong et al. [38]	78/84 (93%)	Mean: 13.4 ± 6.7 days	Median total dose: 160 mg	≈1067 mg
Agarwala et al. [39]	48/48 (100%)	ND	Mean total dose: 841.3 mg	841 mg (as reported)
Dhanasekararaja et al. [40]	22/22 (100%)	Mean: 2.8 weeks	Mean total dose: 811 mg	≈1014 mg

Prednisolone-equivalent doses were estimated using prednisolone 5 mg ≡ methylprednisolone 4 mg ≡ dexamethasone 0.75 mg. Values already reported in prednisolone equivalents are shown unchanged (“as reported”). Velchov et al. [37] values are estimated from the reported regimen and duration NC—not calculable, because the primary study reported only a maximum or daily dose, or did not specify the corticosteroid; ND—no data.

**Table 4:** Quality Assessment of Included Studies.

Study	Tool Used	Score	Quality
Ali et al. [27]	NOS cohort	4/9	Low
Assad et al. [28]	JBI Critical Appraisal Checklist for Case Series	6/10	Moderate
Goel et al. [29]	JBI Critical Appraisal Checklist for Case Series	6/10	Moderate
Imagama et al. [30]	NOS cohort	7/9	High
Koutalos et al. [31]	NOS-xs	7/9	High
Okewunmi et al. [32]	NOS cohort	6/9	Moderate
Sehrawat et al. [33]	NOS-xs	6/9	Moderate
Sharma et al. [34]	NOS-xs	6/9	Moderate
Sujir et al. [35]	NOS cohort	5/9	Moderate
Takashima et al. [36]	NOS cohort	6/9	Moderate
Velchov et al. [37]	NOS cohort	4/9	Low
Seong et al. [38]	NOS cohort	6/9	Moderate
Agarwala et al. [39]	NOS cohort	5/9	Moderate
Dhanasekararaja et al. [40]	NOS cohort	5/9	Moderate
Hogea et al. [41]	NOS cohort	6/9	Moderate

**Table 5:** Methodological heterogeneity of the included studies.

Study	Primary Objective/Design	AVN Staging System	Imaging Modality & Timing	Follow-Up	COVID-19 Severity Definition
Ali et al. [27]	Occurrence; prospective observational	Stage I–III (system NR)	MRI within 6 mo of therapy	NR	Steroid-treated COVID-19 (severity NR)
Koutalos et al. [31]	Occurrence/MRI screening; cross-sectional	NR	MRI hips/shoulders/knees, in-hospital	NR	Hospitalized COVID-19
Takashima et al. [36]	Occurrence/MRI screening; prospective observational	NR	MRI~3 mo post-discharge (26/40)	Serial imaging	Hospitalized COVID-19 pneumonia
Okewunmi et al. [32]	Incidence; retrospective national database	n/a (administrative)	n/a	2016–2021	NR (administrative)
Imagama et al. [30]	Aetiological comparison; multicentre registry	JIC type & stage	Imaging at diagnosis	Registry (short)	NR (not recorded)
Assad et al. [28]	Established-case; retrospective case series	Grade 2–4 (system NR)	MRI at presentation	2021–2022	Hospitalized 47%; ICU 0%
Goel et al. [29]	Established-case; retrospective case series	Steinberg	MRI at presentation	NR	Mild/moderate/severe (institutional)
Sehrawat et al. [33]	Established-case + association; cross-sectional	NR	MRI at presentation	NR	Home/ward/ICU
Sharma et al. [34]	Association within AVN cohort; cross-sectional	NR	Imaging at presentation	NR	NR
Sujir et al. [35]	Established-case; prospective observational	NR	Imaging at presentation	NR	NR
Velchov et al. [37]	Established-case; retrospective	Grade (system NR)	Imaging at orthopaedic assessment	NR	Moderate/severe
Seong et al. [38]	Established-case; retrospective comparative	ARCO	Radiograph/CT/MRI after symptom onset	2021–2023	Lung involvement % (CT)
Agarwala et al. [39]	Established-case; retrospective	NR	Imaging at presentation	NR	NR
Dhanasekararaja et al. [40]	Established-case; prospective observational	JIC type; Kerboul grade	Radiograph + MRI	2020–2021	NR
Hogea et al. [41]	Association within AVN cohort; retrospective comparative	NR	Imaging at presentation	2022–2024	Pulmonary involvement

AVN—avascular necrosis; COVID-19—coronavirus disease 2019; CT—computed tomography; ICU—intensive care unit; MRI—magnetic resonance imaging; NR—not reported; n/a—not appliacble.

## Data Availability

No new data were created or analyzed in this study.

## Appendix A

### Literature Search Strategy

PubMed (MEDLINE):

(“COVID-19” [Mesh] OR “SARS-CoV-2” [Mesh] OR “COVID-19” OR “SARS-CoV-2” OR “coronavirus disease 2019” OR “novel coronavirus”) AND (“Osteonecrosis” [Mesh] OR “avascular necrosis” OR “osteonecrosis” OR “bone necrosis” OR “femoral head necrosis”) AND (“humans” [Mesh])

Embase:

(‘COVID-19’/exp OR ‘SARS-CoV-2/exp OR ‘coronavirus disease 2019’ OR ‘COVID-19’) AND (‘osteonecrosis’/exp OR ‘avascular necrosis’ OR ‘bone necrosis’ OR ‘femoral head necrosis’) AND [humans]/lim

Scopus:

TITLE-ABS-KEY (“COVID-19” OR “SARS-CoV-2” OR “coronavirus disease 2019”) AND TITLE-ABS-KEY (“avascular necrosis” OR “osteonecrosis” OR “bone necrosis” OR “femoral head necrosis”)
